# BMP-7 Treatment Ameliorates PTEN-Akt Mediated Apoptosis and Adverse Cardiac Remodeling in Ponatinib-Induced Cardiotoxicity

**DOI:** 10.3390/ph18121776

**Published:** 2025-11-22

**Authors:** Jonatas M. Rolando, Dinender K. Singla

**Affiliations:** Division of Metabolic and Cardiovascular Sciences, Burnett School of Biomedical Sciences, College of Medicine, University of Central Florida, Orlando, FL 32816, USA; jonatas.demendoncarolando@ucf.edu

**Keywords:** cardiac toxicity, cardiac dysfunction, chronic myeloid leukemia, programmed cell death, tyrosine kinase inhibitors

## Abstract

**Background/Objectives**: Ponatinib (PON) is a potent anticancer drug widely used to treat chronic myeloid leukemia (CML). Although many cancer survivors benefit from such therapies, managing drug-induced side effects, especially cardiotoxicity, remains a major challenge. Despite its prevalence, the exact mechanisms underlying PON-induced cardiotoxicity have not been thoroughly investigated. Additionally, the potential of Bone Morphogenetic Protein 7 (BMP-7) to alleviate these cardiotoxic effects has yet to be explored. **Methods**: To address these essential questions, we conducted a study using C57BL/6 mice. Mice were treated with PON (25 mg/kg cumulative dosage) or a combination of PON and BMP-7 (600 μg/kg), alongside a suitable control group. Heart function was assessed by echocardiography. Different techniques were performed to evaluate the apoptotic pathway. Histological staining was performed to investigate structural changes. **Results**: PON treatment increased apoptotic cell death (increased expression of BAX and caspase-3) in the heart through the PTEN/Akt signaling pathway. Further, PON treatment led to increased cardiac hypertrophy, adverse remodeling, and reduced cardiac function. Importantly, BMP-7 markedly reduced PON-induced apoptosis (increased Bcl2 expression) and its downstream effects. **Conclusions**: These results suggest that BMP-7 might inhibit PON-induced cardiotoxicity. Furthermore, our findings pave the way for future translational studies with BMP-7, which can demonstrate the therapeutic potential of BMP-7 in a clinical setting.

## 1. Introduction

Chronic myeloid leukemia (CML) is characterized by a chromosomal translocation between chromosomes 9 and 22, leading to the fusion of the breakpoint cluster region (BCR) gene and the Abelson murine leukemia viral oncogene homolog (ABL) gene [1]. This fusion produces the constitutively active oncogenic tyrosine kinase known as BCR-ABL [2]. In the United States, an estimated 74,198 individuals are living with CML [3]. Since their introduction, tyrosine kinase inhibitors (TKIs) have significantly improved the survival rates of CML patients by targeting the abnormal tyrosine kinases that cause this disease [4].

Among the available treatments, ponatinib (PON) remains the most effective [5,6]. However, its cardiotoxic side effects pose a considerable challenge for patients [7]. Reports indicate that PON is among the most cardiotoxic TKIs approved by the Food and Drug Administration (FDA) [8]. A recent case report has shown that a CML patient under PON treatment developed cardiomyopathy with decreased ejection fraction (EF) [9]. In this way, cancer survivors are faced with an increased risk of cardiotoxicity due to the very anticancer treatments that saved their lives, threatening not only their quality of life but also raising their risk of cardiac disease and mortality [10,11]. A recent study has revealed a correlation between PON administration and heightened inflammation, which is mediated by the upregulation of the alarmins S100 calcium-binding protein A8 (S100A8) and S100A9 [12]. Furthermore, PON-induced cardiotoxicity has been linked to the activation of the integrated stress response [13]. Additionally, it was observed that PON can reduce autophagy and induce endoplasmic reticulum stress in the heart, playing a significant role in PON-induced cardiotoxicity [14,15]. Despite these findings, the mechanisms underlying PON-induced cardiotoxicity, specifically apoptotic signaling mechanisms, require further investigation.

Apoptosis, a form of programmed cell death, involves a complex interplay of proteins and multiple signaling pathways [16,17]. Particularly, chemotherapy-induced cardiotoxicity can result in the upregulation of the pro-apoptotic protein Bcl2-Associated X-Protein (BAX), triggering downstream caspase-3 activation [18,19,20]. Concurrently, the anti-apoptotic protein B-cell lymphoma 2 (Bcl2) is often downregulated [21]. This process can be regulated by the phosphatase and tensin homolog (PTEN)/Protein kinase B (Akt) signaling pathway [22,23]. However, the relationship between apoptosis, the BAX/Bcl2 regulatory mechanism, and the PTEN-Akt signaling pathway in the context of PON-induced cardiotoxicity remains poorly understood.

Currently, there are no FDA-approved treatments that effectively mitigate the cardiotoxic effects induced by PON-based therapies. Although some studies have proposed potential strategies, such as the simultaneous administration of neuregulin-1β or glucocorticoid medications, these options are insufficient to address the issue of PON-induced cardiotoxicity [12,24]. In light of this, we propose Bone Morphogenetic Protein 7 (BMP-7)—a growth factor and FDA-approved therapy for bone regeneration [25,26]—as a potential solution for PON-induced cardiotoxicity. BMP-7 has recently shown promise as a therapeutic candidate, with research indicating its ability to counteract pre-diabetic and diabetic cardiomyopathy [27,28]. This raises the critical question: can BMP-7 extend its cardioprotective effects to combat PON-induced cardiotoxicity?

Our study aims to achieve three primary objectives: First, we will delineate the critical role of PON in inducing apoptotic cell death in the heart, along with its subsequent effects on cardiac hypertrophy, fibrosis, and overall function. Second, we will investigate the molecular mechanisms underlying this apoptotic process. Ultimately, we aim to examine whether BMP-7 treatment can effectively counteract apoptotic cell death, modulate the associated mechanisms, and reverse adverse cardiac remodeling while enhancing cardiac function.

## 2. Results

### 2.1. Effect of BMP-7 on Mice Heart Weight Following PON Treatment

**Figure 1:**
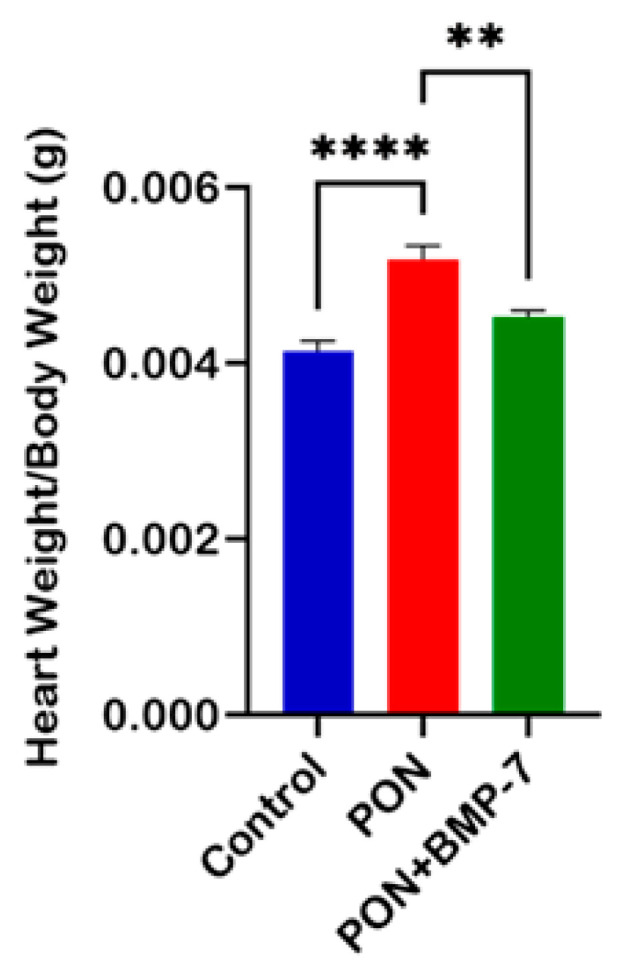
Effect of BMP-7 on mice heart weight following PON treatment. Heart-to-body-weight ratios on D19 across all groups (*n* = 6/group), *n* = number of animals. Data is presented as mean ± standard error of the mean (SEM). Statistical analysis: one-way analysis of variance (ANOVA) with Tukey’s post hoc test; ** *p* < 0.01, **** *p* < 0.0001.

To assess the impact of BMP-7 on cardiac muscle in the context of PON administration, we computed the heart weight to body weight ratio. We showed a significant increase (*p* < 0.0001) in this ratio within the PON group compared to the control group (Figure 1). Notably, the data also indicated a substantial decrease (*p* < 0.01) in the BMP-7 group relative to the PON group, suggesting that BMP-7 treatment effectively mitigates the PON-induced increase in heart weight.

### 2.2. BMP-7 Treatment Inhibits PON-Induced Apoptosis in the Heart

**Figure 2:**
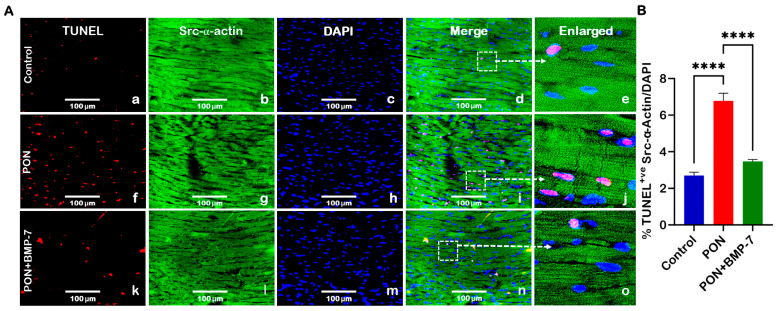
BMP-7 Treatment inhibits PON-induced apoptosis in the heart. (**A**) Representative images of heart sections co-stained for TUNEL (red; **a**,**f**,**k**), Src-α-actin (green; **b**,**g**,**l**), and 4′,6-diamidino-2-phenylindole (DAPI) (blue; **c**,**h**,**m**). Merged (**d**,**i**,**n**) and magnified panels (section highlighted in dotted white boxes and arrows; **e**,**j**,**o**) highlight apoptotic cardiomyocytes. Scale bar = 100 µm. (**B**) Quantification of TUNEL-positive nuclei in all groups (*n* = 6/group). Data is presented as mean ± SEM. Statistical analysis: one-way ANOVA with Tukey’s test; **** *p* < 0.0001.

To investigate the effects of PON administration on cardiac apoptosis and the potential protective role of BMP-7, we utilized terminal deoxynucleotidyl transferase dUTP nick end labeling (TUNEL) staining. TUNEL staining detects DNA fragmentation from apoptotic cell death [29]. Cardiomyocytes were co-stained for TUNEL in red and cardiac sarcomeric alpha-actin (Src-α-actin) in green. Our findings revealed a marked increase in TUNEL-positive nuclei (indicative of apoptotic cells) in the PON group (f–j) compared to the control group (a–e). Conversely, the BMP-7 treatment (k–o) led to a significant reduction in TUNEL-positive nuclei when compared to the PON group (Figure 2A). Quantitative analysis (Figure 2B) further confirmed a significant increase (*p* < 0.0001) in the number of TUNEL-positive cardiomyocytes in the PON group compared to the control. These results indicate that PON administration induces apoptosis in cardiomyocytes, a process that can be rescued by BMP-7 treatment.

### 2.3. BMP-7 Treatment Reduces the Pro-Apoptotic Marker BAX in PON-Administered Hearts

**Figure 3:**
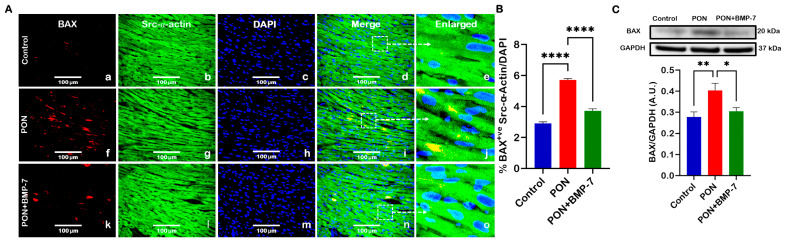
BMP-7 treatment reduces the pro-apoptotic marker BAX in PON-administered hearts. (**A**) Immunohistochemical images showing BAX (red; **a**,**f**,**k**), Src-α-actin (green; **b**,**g**,**l**), and DAPI (blue; **c**,**h**,**m**) co-localization (merged; **d**,**i**,**n**) in heart sections. White dotted boxes and arrows denote enlarged sections (**e**,**j**,**o**) of merged images. Scale bar = 100 µm. (**B**) Quantitative analysis of BAX-positive cardiomyocytes (*n* = 6/group). (**C**) Representative blots and densitometric quantification of BAX protein expression (*n* = 6–7/group; arbitrary units (A.U.)). Data is presented as mean ± SEM. Statistical analysis: one-way ANOVA with Tukey’s test; * *p* < 0.05, ** *p* < 0.01, **** *p* < 0.0001.

To further validate TUNEL-mediated apoptosis, we examined the apoptotic pathway marker BAX using immunohistochemistry (IHC) and Western blotting (WB). Representative IHC images (Figure 3A) illustrated that BAX (red) and Src-α-actin (green) co-staining had a higher number of BAX-positive cardiomyocytes in the PON-treated mice (f–j) compared to the control group (a–e). Notably, the BMP-7 treatment group (k–o) exhibited a decreased number of BAX-positive cardiomyocytes when juxtaposed with the PON group. IHC quantification (Figure 3B) corroborated that the pro-apoptotic marker BAX was significantly elevated (*p* < 0.0001) in the cardiomyocytes of the PON group, while this increase was notably diminished following BMP-7 treatment. Additionally, our WB analysis (Figure 3C) indicated a significant (*p* < 0.01) rise in BAX protein expression in the hearts of the PON group, which was inhibited in the BMP-7 treatment group (*p* < 0.05), suggesting that PON can initiate the intrinsic apoptotic pathway through an increase in BAX expression. This response is mitigated by BMP-7 treatment.

### 2.4. BMP-7 Treatment Reduces the Pro-Apoptotic Marker Caspase-3 in PON-Administered Hearts

**Figure 4:**
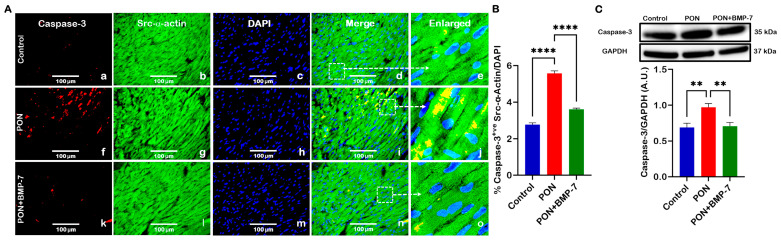
BMP-7 treatment reduces the pro-apoptotic marker caspase-3 in PON-administered hearts. (**A**) Representative IHC photomicrographs of caspase-3 (red; **a**,**f**,**k**), Src-α-actin (green; **b**,**g**,**l**), and DAPI (blue; **c**,**h**,**m**). Merged (**d**,**i**,**n**) and magnified panels (section highlighted in dotted white boxes and arrows; **e**,**j**,**o**) show caspase-3-positive cardiomyocytes. Scale bar = 100 µm. (**B**) IHC quantification of caspase-3 expression in cardiomyocytes (*n* = 6/group). (**C**) Western blots and quantitative analysis of caspase-3 protein levels (*n* = 6–7/group; A.U.). Data is presented as mean ± SEM. Statistical analysis: one-way ANOVA with Tukey’s test; ** *p* < 0.01, **** *p* < 0.0001.

BAX can drive downstream activation of caspase-3 through the release of cytochrome c from mitochondria [30]. Therefore, we aimed to understand whether PON treatment could induce increased caspase-3 expression. Our IHC photomicrographs (Figure 4A) revealed co-staining of caspase-3 (red) and Src-α-actin (green). Notably, we observed an increased colocalization of caspase-3 and Src-α-actin in the PON group (f–j) compared to the control group (a–e). In contrast, the BMP-7 group (k–o) exhibited decreased caspase-3 expression relative to the PON group. Additionally, quantitative analysis of the IHC demonstrated that caspase-3 levels (Figure 4B) were significantly upregulated (*p* < 0.0001) in the cardiomyocytes of the PON group compared to the control group. Interestingly, BMP-7 treatment resulted in a significant downregulation (*p* < 0.0001) of caspase-3 expression compared to the PON group. Furthermore, our WB analysis (Figure 4C) showed that caspase-3 protein levels were significantly elevated (*p* < 0.01) in the PON group compared to the controls. In contrast, the BMP-7 group showed a significant decrease (*p* < 0.01) in caspase-3 expression relative to the PON group. Overall, these findings suggest that PON enhances caspase-3 expression in cardiomyocytes, promoting apoptotic cell death, an effect that can be attenuated by BMP-7 treatment.

### 2.5. BMP-7 Treatment Enhances the Anti-Apoptotic Protein Bcl2 in PON-Administered Hearts and Decreases BAX/Bcl2 Ratio

**Figure 5:**
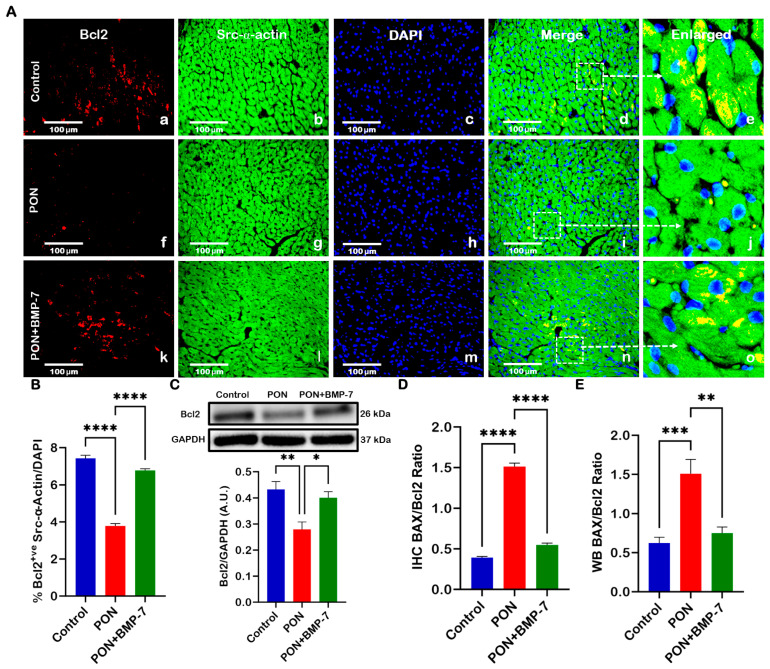
BMP-7 treatment enhances the anti-apoptotic protein Bcl2 in PON-administered hearts and decreases BAX/Bcl2 Ratio. (**A**) Representative heart sections co-stained for Bcl-2 (red; **a**,**f**,**k**), Src-α-actin (green; **b**,**g**,**l**), and DAPI (blue; **c**,**h**,**m**). Merged (**d**,**i**,**n**) and magnified panels (section highlighted in dotted white boxes and arrows; **e**,**j**,**o**) reveal Bcl2-positive cardiomyocytes. Scale bar = 100 µm. (**B**) Quantitative analysis of Bcl-2-positive cells (*n* = 6/group). (**C**) Representative blots and densitometric analysis of Bcl-2 protein (*n* = 6/group; A.U.). (**D**,**E**) BAX/Bcl-2 ratios derived from IHC and WB (Figure 3 and Figure 5) analyses (*n* = 6/group; A.U.). Data is presented as mean *±* SEM. Statistical analysis: one-way ANOVA with Tukey’s test; * *p* < 0.05, ** *p* < 0.01, *** *p* < 0.001, **** *p* < 0.0001.

We also investigated the expression of the anti-apoptotic marker Bcl2. Heart sections (Figure 5A) were co-stained for Bcl2 (red) and Src-α-actin (green). Our analysis revealed a lower number of Bcl2-positive cardiomyocytes in the PON group (f–j) compared to the control group (a–e). In contrast, the BMP-7 group (k–o) exhibited a higher presence of Bcl2-positive cardiomyocytes relative to the PON group. Quantitative analysis (Figure 5B) further supported these observations, showing a significant reduction (*p* < 0.0001) in Bcl2-positive cells in the PON group, while BMP-7 treatment led to a significant increase (*p* < 0.0001). To corroborate these findings, we conducted Western blotting analysis (Figure 5C), which revealed a significant decrease (*p* < 0.01) in Bcl-2 protein expression in the PON group compared to the controls. Conversely, the BMP-7-treated mice displayed a significant increase (*p* < 0.05) in Bcl2 protein levels when compared to the PON group. This data indicates that BMP-7 treatment can rescue cardiomyocytes from apoptotic cell death by enhancing Bcl2 expression.

The BAX/Bcl2 ratio is a key indicator of the balance between pro-apoptotic and anti-apoptotic proteins, reflecting a cell’s susceptibility to apoptosis [31]. To evaluate this balance under PON treatment, we calculated the BAX/Bcl2 ratio from our IHC (Figure 5D) and WB (Figure 5E) data. Our IHC results demonstrated a significant increase (*p* < 0.0001) in the BAX/Bcl-2 ratio for the PON group compared to the control group. In contrast, the BMP-7 group exhibited a significant reduction (*p* < 0.0001) in this ratio compared to the PON group. Further analysis of the WB data confirmed these trends, showing a significant increase (*p* < 0.001) in the BAX/Bcl2 ratio in the PON group compared to the control group, along with a notable decrease (*p* < 0.01) in the BMP-7 group when compared to the PON group. These results suggest that PON administration increases the susceptibility of cardiomyocytes to apoptotic cell death, ultimately leading to cardiotoxicity.

### 2.6. BMP-7 Inhibits PON-Induced Cardiotoxicity via PTEN/Akt Signaling Pathway

**Figure 6:**
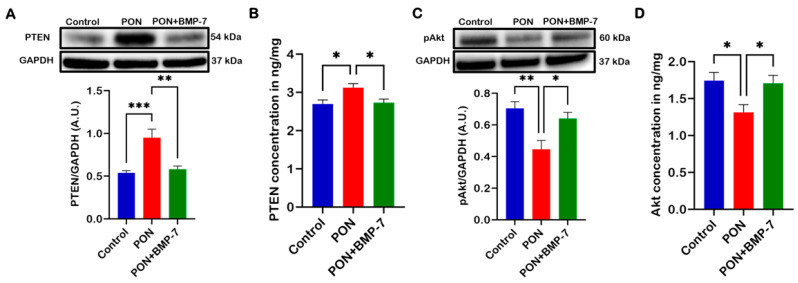
BMP-7 inhibits PON-induced cardiotoxicity via PTEN/Akt signaling pathway. (**A**,**C**) Western blots and quantitative analyses of PTEN (*n* = 6–7/group; A.U.) and pAkt (*n* = 6/group; A.U.) protein levels in cardiac tissue. (**B**,**D**) ELISA quantification of PTEN and Akt levels in 25 µg of protein from cardiac tissue (*n* = 6/group). Data is presented as mean ± SEM. Statistical analysis: one-way ANOVA with Tukey’s test; * *p* < 0.05, ** *p* < 0.01, and *** *p* < 0.001.

The PTEN/Akt signaling pathway is well-known for its role in cardiomyocyte apoptosis [29]. PTEN is capable of inducing cell cycle arrest and apoptosis, whereas Akt promotes cell proliferation and suppresses apoptosis [32]. To investigate the underlying signaling mechanisms behind PON-induced cardiotoxicity and its inhibition by BMP-7, we conducted Western blot analyses and enzyme-linked immunosorbent assays (ELISA). Our WB analysis (Figure 6A) revealed that PTEN expression was significantly increased (*p* < 0.001) in the PON group when compared to the control. Conversely, the BMP-7 group exhibited a significant decrease (*p* < 0.01) in PTEN protein expression relative to the PON group. Similarly, the ELISA results showed a significant increase (*p* < 0.05) in PTEN levels (Figure 6B) in the PON group compared to the control. Notably, the BMP-7 group also showed a significant decrease (*p* < 0.05) in PTEN levels compared to the PON group.

Furthermore, our WB analysis of phosphorylated Akt (pAkt) (Figure 6C) demonstrated a significant decrease (*p* < 0.01) in expression in the PON group compared to the control. At the same time, treatment with BMP-7 resulted in a substantial increase (*p* < 0.05) in pAkt expression relative to the PON group. ELISA analysis corroborated these findings, showing significantly reduced Akt levels (Figure 6D) in the PON group compared to the control (*p* < 0.05). Interestingly, the BMP-7 group exhibited significantly elevated Akt levels (*p* < 0.05) compared to the PON group. These results suggest that PON induces cardiomyocyte apoptosis via the PTEN/Akt signaling pathway, leading to the onset of PON-induced cardiotoxicity, which can be mitigated by BMP-7 inhibition.

### 2.7. BMP-7 Ameliorates PON-Induced Cardiac Hypertrophy and Fibrosis

**Figure 7:**
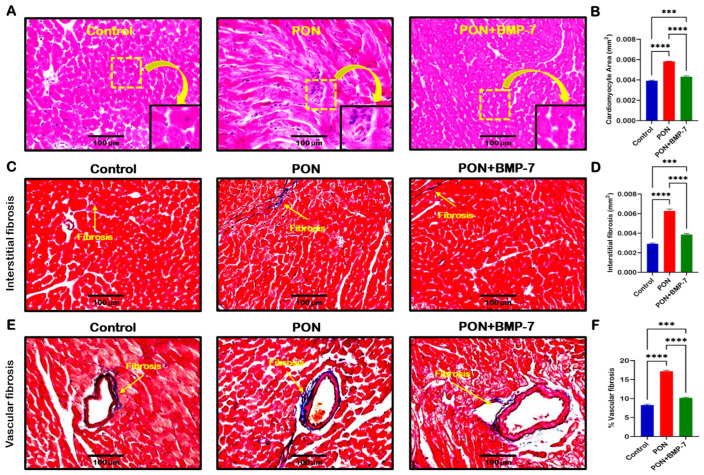
BMP-7 ameliorates PON-induced cardiac hypertrophy and fibrosis. (**A**) Representative images of H&E-stained sections showing cardiomyocyte hypertrophy and immune cell infiltration in the PON group. Yellow dotted boxes and arrows denote enlarged sections of representative images. Scale bar = 100 µm. (**B**) Quantification of cardiomyocyte area (*n* = 6/group). (**C**,**E**) Masson’s trichrome staining of interstitial and vascular fibrosis (blue areas indicated by yellow arrows). Scale bar = 100 µm. (**D**,**F**) Quantification of interstitial and vascular fibrosis (*n* = 6/group). Data is presented as mean ± SEM. Statistical analysis: one-way ANOVA with Tukey’s test; *** *p* < 0.001 and **** *p* < 0.0001.

Cardiac apoptosis has been linked to the development of adverse cardiac structural remodeling [33]. Thus, we aimed to investigate whether PON-induced apoptosis contributes to increased cardiac hypertrophy and fibrosis. First, we conducted hematoxylin and eosin (H&E) staining. Representative images of H&E staining (Figure 7A) demonstrated that PON-treated mice exhibited a larger cardiomyocyte area and greater immune cell infiltration compared to control mice. In contrast, the BMP-7 treatment group showed a reduced cardiomyocyte area relative to the PON-treated group. Our quantitative analysis (Figure 7B) confirmed a significant increase in cardiac hypertrophy in the PON group compared to the control group (*p* < 0.0001). In contrast, the BMP-7 group exhibited a substantial reduction in hypertrophy compared to the PON group.

Next, we performed Masson’s trichrome staining to analyze cardiac fibrosis under PON treatment. Our representative images showed that the PON group had increased fibrosis, as indicated by the blue area, in both interstitial (Figure 7C) and vascular fibrosis (Figure 7E), compared to the control group, which was reduced upon BMP-7 treatment. Furthermore, our quantitative data confirmed a significant increase (*p* < 0.0001) in collagen deposition in both interstitial and vascular fibrosis in the PON group compared to the control (Figure 7D,F). BMP-7 treatment showed a significant (*p* < 0.0001) decrease in interstitial and vascular collagen deposition when compared to PON group. Overall, these results suggest that PON-induced apoptotic cell death can drive increased hypertrophy and fibrosis, furthering PON-induced cardiotoxicity progression and adverse structural alterations, which can be improved with BMP-7 treatment.

### 2.8. BMP-7 Improves Heart Function in PON-Induced Cardiotoxicity

**Figure 8:**
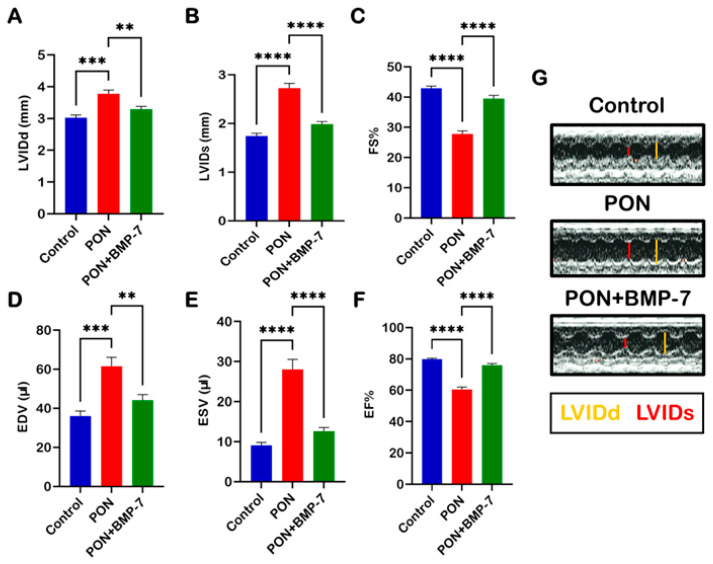
BMP-7 improves heart function in PON-induced cardiotoxicity. Echocardiographic evaluation on D19 across all groups. (**A**–**F**) Quantitative analysis of different echocardiographic parameters (*n* = 7–8/group). (**G**) Representative M-mode echocardiographic images illustrating improved ventricular dimensions following BMP-7 treatment. Data is presented as mean ± SEM. Statistical analysis: one-way ANOVA with Tukey’s test; ** *p* < 0.01, *** *p* < 0.001, and **** *p* < 0.0001.

It is well established that structural remodeling can induce heart failure [34]. Therefore, we investigated the impact of PON-induced adverse cardiac remodeling on cardiac function. To assess this, we examined left ventricular function across all groups using echocardiography. We measured several parameters, including left ventricular internal dimension during diastole (LVIDd), left ventricular internal dimension during systole (LVIDs), fractional shortening (FS), end-diastolic volume (EDV), end-systolic volume (ESV), and ejection fraction (EF) (Figure 8A–F). Our data indicate that PON administration is associated with impaired left ventricular function, demonstrated by a significant increase in LVIDd (*p* < 0.001), LVIDs (*p* < 0.0001), EDV (*p* < 0.001), and ESV (*p* < 0.001). Additionally, key indicators of cardiac dysfunction, namely left ventricular (LV)-FS% (*p* < 0.0001) and LV-EF% (*p* < 0.0001), were markedly reduced in the PON group compared to controls, suggesting a decline in cardiac performance. Notably, BMP-7 treatment significantly restored cardiac function relative to the PON group. Our M-mode images (Figure 8G) further corroborate these findings, illustrating increased LVIDd and LVIDs in the PON group. Collectively, these results suggest that PON-induced cardiotoxicity can induce cardiac dysfunction, which may be ameliorated through BMP-7 treatment.

## 3. Discussion

PON is a third-generation TKI specifically designed to overcome the resistance of the T315I mutation to other TKIs in patients with CML [35,36]. Although PON is effective against leukemia, its clinical use is restricted by cardiotoxic effects like arrhythmia, hypertension, thrombosis, and heart failure [37]. Recent studies have shown that PON-induced cardiotoxicity is related to increased inflammation, senescence, and intracellular stress [12,13,14,15]. In this study, we (a) explored the pivotal role of PON in triggering apoptotic cell death within the heart, (b) established the PON-induced apoptosis, and underlying molecular mechanism via PTEN-Akt pathway leading to cardiac dysfunction in PON-induced cardiotoxicity; finally, (c) we provided a strong evidence on how BMP-7 treatment can counteract apoptotic cell death and associated signaling mechanisms in the heart with improved cardiac function.

Apoptosis is a programmed cell death with interconnected pathways. Death receptors mediate the extrinsic pathway, while the intrinsic pathway is mediated by internal protein signaling [38]. To confirm PON-induced apoptosis, TUNEL staining was performed, showing increased TUNEL-positive nuclei in PON-treated mice. These results are in confirmation with other studies, where it was observed that PON indeed increases TUNEL-positive nuclei in the heart [13,39].

Furthermore, BAX is an important pro-apoptotic protein that can oligomerize when stimulated by truncated BH3-interacting-domain death agonist (tBID). After oligomerization and translocation to the outer mitochondrial membrane, BAX can enhance the permeability of the membrane, leading to apoptotic cell death [38,40,41]. Bcl2 is an anti-apoptotic protein that can bind to BAX and prevent the outer mitochondrial membrane from leaking, inhibiting apoptosis [42,43]. Previously, it was observed that zebrafish treated with PON had higher expression of BAX in their hearts [24]. Nevertheless, we reported for the first time an increased expression of BAX and a decreased expression of Bcl2, leading to an augmented BAX/Bcl2 ratio in the hearts of PON-administered mice.

After the outer mitochondrial membrane is permeable, there is an overflow of cytochrome c into the cytoplasm, which leads to activation of caspase-3 and apoptotic cell death [44]. In our study, we observed increased caspase-3 expression in the hearts of PON mice. This finding aligns with a recent study showing that PON-treated human-induced pluripotent stem cell-derived cardiomyocytes (hiPSC-CMs) exhibited increased caspase-3 expression [13]. These results revealed that PON can induce increased expression of pro-apoptotic proteins and DNA fragmentation. Further, it stimulates decreased expression of anti-apoptotic proteins, leading to cardiotoxicity through apoptotic cell death.

PTEN is responsible for the dephosphorylation of phosphatidylinositol 3,4,5-trisphosphate (PIP3). This inhibits Akt phosphorylation, promoting cell cycle arrest and cell death [45,46]. Therefore, to understand the underlying signaling mechanisms of PON-induced cardiotoxicity, we investigated the PTEN/Akt pathway. Our novel work is the first to demonstrate that PON administration results in increased PTEN expression in the heart. Additionally, we observed decreased Akt expression, which confirms the findings in the hearts of PON-treated zebrafish [24]. Therefore, our data suggests that PON-induced cardiotoxicity can be mediated through the PTEN/Akt signaling pathway.

There is evidence linking cardiac apoptosis with the development of cardiac hypertrophy and fibrosis, ultimately leading to heart failure [47,48]. In the PON phase II clinical trials, left ventricular hypertrophy was one of the most frequently reported heart failure events [49]. We are the first to show that PON-treated mice have increased heart weight and cardiomyocyte area. Additionally, our study demonstrated that PON-treated mice hearts had increased interstitial and vascular fibrosis. This finding aligns with a recent study, which showed that PON-treated apolipoprotein E (ApoE) knockout mice fed a high-fat diet exhibited increased cardiac fibrosis [12]. Additionally, PON caused a marked decline in cardiac function, highlighting the impact of these pathological changes on heart performance. Similar results were observed in another study where mice treated with PON had reduced LV-EF% [14,50]. Therefore, PON-induced cardiotoxicity can lead to the development of adverse cardiac remodeling, followed by cardiac dysfunction.

Based on current literature, no treatments are available to ameliorate PON-induced cardiotoxicity. BMP-7 is known for its anti-inflammatory properties, leading to the investigation of BMP-7 as a treatment for different diseases [28,51,52]. Moreover, BMP-7 stimulates downstream responses through the suppressor of mothers against decapentaplegic (SMAD) and mitogen-activated protein kinase (MAPK) pathways [53,54]. Human pharmacokinetic data for BMP-7 are limited, but a pre-clinical study showed a short half-life of 30 min after intravenous (i.v.) administration. Additionally, fractions of the administered dose reach BMP-7’s receptor-bearing organs, such as the kidney, heart, brain, and bones [55]. Moreover, BMP-7 can exert pleiotropic effects in different organs. BMP-7 administration reduced renal fibrosis, provided neuroprotection in ischemic stroke injury and Parkinson’s disease, and promoted bone regeneration, with no reported off-target toxicity [56,57,58,59]. While BMP-7 has undergone clinical evaluation in other contexts [60,61], its role in mitigating PON-induced cardiotoxicity remains to be elucidated.

In this study, BMP-7 treatment reduced TUNEL-positive nuclei, lowered BAX and caspase-3 expression, and increased Bcl-2 levels in heart tissue compared with the PON group. Moreover, we observed a decrease in the BAX/Bcl2 ratio and PTEN expression, in addition to an increase in Akt expression, in the BMP-7 group compared to the PON group. Finally, BMP-7 treatment attenuated the PON-induced increase in heart weight, reduced adverse cardiac remodeling, and restored cardiac function, with LV-FS% and LV-EF% comparable to control levels. Our results are consistent with previous reported studies showing that BMP-7 can rescue cardiomyocytes from apoptosis [27,62], ameliorate adverse cardiac remodeling, and restore cardiac function in diabetic and pre-diabetic mice [27,28]. Therefore, our findings demonstrate that BMP-7 effectively mitigates PON-induced cardiotoxicity, providing a strong scientific and methodological basis for advancing this preclinical discovery toward clinical translation.

Our data provides strong evidence of BMP-7’s potential in reducing PON-induced cardiotoxicity; however, the study has some limitations. Although BMP-7 is approved by the FDA for bone regeneration treatment [25], its systemic use still requires clinical trial validation. Also, our current study is limited to a shorter time point (D19), suggesting the requirement for further research for additional time points and long-term follow-up studies. In addition, our study is based on preclinical mouse data, providing a strong foundation for future in vitro investigations using human cardiomyocytes to further enhance translational relevance. Nevertheless, our study reveals novel mechanisms of PON-induced cardiotoxicity and demonstrates the cardioprotective potential of BMP-7 in a preclinical model.

## 4. Materials and Methods

### 4.1. Experimental Design

**Figure 9:**
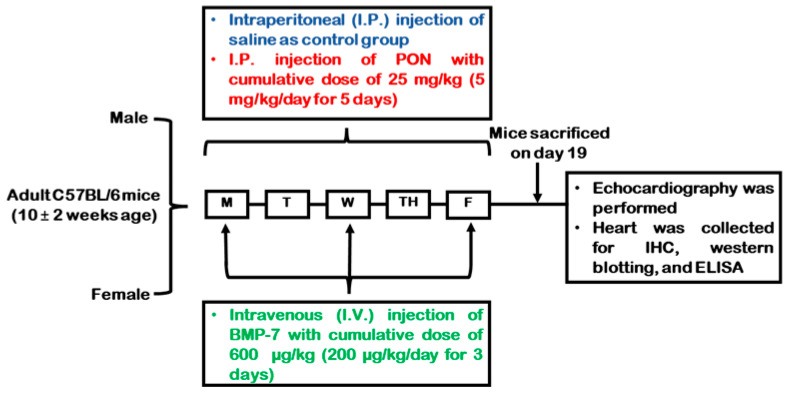
Experimental design schematics. Injection schedule of control, PON, and PON+BMP-7 groups. On day 19 (D19) after the first injection, mice underwent echocardiography, followed by euthanasia and heart collection.

All animal procedures were performed in accordance with the approval of the Institutional Animal Care and Use Committee (IACUC) of the University of Central Florida (UCF), following guidelines established by the National Institutes of Health (NIH). Throughout the treatment period, mice were closely monitored for signs of illness or distress such as lethargy, reduced appetite, weight loss, poor grooming, diminished interaction, hunched posture, dehydration, swelling, and skin or fur changes. In the current study, we did not observe signs of distress in any experimental PON-treated mice. All mice were euthanized humanely following the approved IACUC protocol. PON was obtained from Selleckchem (Houston, TX, USA; Cat. #S1490), while BMP-7 was obtained from Bioclone (San Diego, CA, USA; Cat. #PA-0401). C57BL/6J (Jackson Labs, Bar Harbor, ME, USA) mice of 10 ± 2 weeks of age, were divided into 3 different groups (*n* = 9/group; males and females); control (0.9% saline for five consecutive days), PON (5 mg/kg/day through intraperitoneal (i.p.) injection for five straight days with a cumulative dose of 25 mg/kg), and PON+BMP-7 (200 μg/kg/day through intravenous (i.v.) injection in three alternate days with a cumulative dose of 600 μg/kg), following protocol previously used (Figure 9) [63]. Mice’s body weights were recorded before the first injection and at D19. The mice were subjected to echocardiography before euthanasia under 4% isoflurane for 10 min, followed by cervical dislocation. Hearts were harvested and washed with 1X phosphate-buffered saline (PBS), weighed, and transversely divided into two parts. The top part was stored in a −80 °C freezer for molecular analysis, and the bottom part was stored in 4% paraformaldehyde (PFA) for histological analysis.

### 4.2. Heart Weight Ratio

The ratio of heart to body weight was analyzed by normalizing heart weight to final body weight, as described previously [64]. The data were analyzed, showing the difference in normalized heart weight.

### 4.3. Tissue Processing

Heart tissues were stored for 24 h in 4% PFA, washed three times in 1X PBS, and then stored in 70% ethanol for an additional 24 h. Next, heart tissues were processed in a Leica tissue processor (Leica, Allendale, NJ, USA), using ethanol solutions of different percentages, followed by CitriSolv (Decon Laboratories Inc., King of Prussia, PA, USA) and paraffin. Heart tissues were embedded in paraffin using a tissue embedder (Sakura Finetek, Torrance, CA, USA). The paraffin blocks were then sectioned into 5-μm sections using a microtome (Thermo Fisher Scientific, Waltham, MA, USA) and placed on ColorFrost™ Plus slides (Thermo Fisher Scientific) for all histological analysis, including TUNEL staining.

### 4.4. Terminal Deoxynucleotidyl Transferase dUTP Nick End Labeling (TUNEL) Staining

TUNEL staining was performed using In Situ Cell Death Detection Kit, TMR red (Millipore Sigma, Burlington, MA, USA; Cat. #12156792910), as described previously by us [63,65]. Heart sections were deparaffinized and permeabilized with proteinase K (25 μg/mL in 100 mM tris-hydrochloride) for 15 min, followed by TUNEL staining. After that, cardiomyocytes were stained with cardiac Src-α-actin (Sigma-Aldrich, St. Louis, MO, USA; Cat. #A2172), using M.O.M.^®^ (Mouse on Mouse) Immunodetection Kit (Vector Laboratories, Newark, CA, USA). Then, sections were mounted with DAPI (Vector Laboratories; Cat. #H-1200). Quantification of TUNEL-positive cells (apoptotic nuclei) was done using 20× images taken with a Keyence microscope (BZ-X810-Keyence, Itasca, IL, USA), and quantified with ImageJ 1.39o (NIH, Bethesda, MD, USA). While 40× images were taken for representative purposes.

### 4.5. Immunohistochemistry (IHC) Staining

Double IHC was performed on heart tissue sections using the M.O.M. kit, following the supplier’s instructions as previously described [66,67]. In brief, heart sections were stained for cardiac Src-α-actin (1:250 *v*/*v* dilution in M.O.M. Protein Concentrate), followed by blocking with 10% normal goat serum (NGS) (Vector Laboratories) and incubation with primary antibodies (1:250 *v*/*v* dilution in 10% NGS): BAX (Santa Cruz Biotechnology, Dallas, TX, USA; Cat. #sc-493), caspase-3 (Santa Cruz Biotechnology; Cat. #sc-7148), and Bcl2 (Santa Cruz Biotechnology; Cat. #sc-492). After that, sections were washed with 1X PBS and incubated with Alexa Fluor^®^ 568 goat anti-rabbit antibody (Invitrogen, Carlsbad, CA, USA; Cat. #A11011). Then, the sections were washed and mounted with DAPI. Quantification and representative images were performed as aforementioned in TUNEL staining. We used the percentage of BAX-positive cardiomyocytes divided by the rate of Bcl2-positive cardiomyocytes to calculate BAX/Bcl2 IHC ratio following the protocol previously described by us [63].

### 4.6. Western Blotting (WB)

Heart tissues were homogenized in radioimmunoprecipitation assay buffer (RIPA), and protein concentration was estimated using the Bio-Rad protein assay (Bio-Rad, Hercules, CA, USA). The samples were then run on SDS-PAGE gels, followed by Western blotting as previously described [54]. Briefly, 25 µg of protein was loaded into Bolt gels (4–12%, Invitrogen) and run at 200 V for 22 min in a Mini Gel Tank (Invitrogen) using a PowerEase™ unit (Invitrogen). The gels were transferred to polyvinylidene difluoride (PVDF) membranes for 7 min using the iBlot™ 2 system (Invitrogen). Then, 5% non-fat milk (NFM) or 5% bovine serum albumin (BSA for phosphorylated proteins) prepared in 1X TBS-T (tris-buffered saline, 0.1% Tween-20) was used to block the membranes for 1 h at room temperature (RT), followed by incubation with primary antibodies (1:1000 *v*/*v* dilution in 5% NFM or 5% BSA): BAX (Cell Signaling, Danvers, MA, USA; Cat. #2772S), caspase-3 (Cell Signaling; Cat. #9662S), Bcl2 (Cell Signaling; Cat. #3498S), PTEN (Cell Signaling; Cat. #9559L), pAkt (Cell Signaling; Cat. #4058L), and glyceraldehyde-3-phosphate dehydrogenase (GAPDH) (Cell Signaling; Cat. #5174), for overnight at 4 °C. After that, the PVDF membranes were washed with 1X TBS-T, before incubation with secondary antibody Anti-rabbit IgG, HRP-linked Antibody (Cell Signaling; Cat. #7074) for 1 h at RT (1:1000 *v*/*v* dilution in 5% NFM or 5% BSA). Then, the membranes were washed again with 1X TBS-T and immersed in Pierce™ ECL (Thermo Fisher Scientific) before developing the image with Azure Sapphire™ Biomolecular Imager (Azure Biosystems, Dublin, CA, USA). ImageJ 1.39o (NIH) was used to perform densitometric analysis, with band intensity being normalized by GAPDH and expressed as an arbitrary unit. BAX densitometric intensity divided by Bcl2 densitometric intensity was used to calculate the WB BAX/Bcl2 ratio. All original Western blot images from Figure 3, Figure 4, Figure 5 and Figure 6 are available in Supplementary Materials (S1).

### 4.7. Enzyme-Linked Immunosorbent Assay (ELISA)

ELISA was performed following the protocol previously described by us [63], using PTEN (MyBioSource, San Diego, CA, USA; Cat. # MBS3806814) and Akt (MyBioSource; Cat. # MBS288382) ELISA kits. Briefly, 25 μg of the protein homogenate from heart tissues was used to determine the levels of PTEN and Akt, following the manufacturer’s instructions. Optical density was measured at 450 nm using a Multiskan FC Microplate Photometer (Thermo Fisher Scientific).

### 4.8. Histological Staining

H&E staining was performed to assess cardiac hypertrophy, following the protocol previously described by us [23]. In brief, heart sections were stained with hematoxylin (Epredia, Kalamazoo, MI, USA; Cat. #7211), acid alcohol 1% (Poly Scientific R&D Corp., Bay Shore, NY, USA; Cat. #S104), bluing reagent (Thermo Fisher Scientific; Cat. #7301), and eosin (Epredia; Cat. #6766008). Then, the sections were dehydrated with a series of ethanol and xylene, followed by mounting with Permount™ (Thermo Fisher Scientific; Cat. #SP15-100). Cardiomyocytes’ cytoplasm was stained pink, while nuclei were stained blue. The quantification of cardiomyocyte size (mm^2^) was performed with ImageJ software version 1.39o (NIH) as mentioned before.

Masson’s trichrome staining was performed to evaluate interstitial and vascular fibrosis, following the protocol previously described by us [67]. In short, following rehydration, heart sections were first incubated in Bouin’s fixative (Poly Scientific R&D Corp.; Cat. #S129) for 45 min at 62.3 °C. Then, sections were stained with a mixture of Weigert’s iron hematoxylin solutions A&B (Poly Scientific R&D Corp.; Cat. #S216BA, #S216BB), Biebrich scarlet-acid fuchsin solution (Poly Scientific R&D Corp.; Cat. #S125), 5% phosphotungstic/phosphomolybdic acid solution (Thermo Fisher Scientific; Cat. #A248-100, #A237-100), aniline blue (Poly Scientific R&D Corp.; Cat. #S116), and 1% glacial acetic acid (Thermo Fisher Scientific; Cat. #A491-212). Next, the sections were dehydrated and mounted with Permount™. In Masson’s trichrome staining, the cytoplasm of cardiomyocytes was stained red, while nuclei were stained black. The quantification was performed as aforementioned. The interstitial fibrosis was investigated by measuring total fibrotic area (mm^2^) in tissue (collagen deposition in blue), while the percentage of vascular fibrosis was examined according to the formula: (vascular fibrosis area/vessel area) × 100.

### 4.9. Echocardiography

On D19, 2D echocardiography was performed using a Sonos 5500 ultrasound system (Hewlett-Packard, Andover, MA, USA) with a 15 MHz transducer (Philips, Cambridge, MA, USA), according to the protocol described by us [22,67]. M-mode images were recorded, and different cardiac measurements were analyzed to assess cardiac function.

### 4.10. Statistical Analysis

Statistical significance between groups was observed via one-way ANOVA, followed by Tukey’s post hoc test, using GraphPad Prism software version 10.1.0 (GraphPad Software, Boston, MA, USA). Values are displayed as mean ± standard error of the mean, with *p* < 0.05 being considered statistically significant.

## 5. Conclusions

In conclusion, our findings compellingly illustrate that PON has the potential to induce cardiotoxicity primarily through apoptotic cell death mechanisms. This is evidenced by significant increases in TUNEL staining, as well as elevated levels of BAX and caspase-3 expression, alongside a notable decrease in Bcl-2 expression within the hearts of mice. Furthermore, we have elucidated that the PTEN/Akt signaling pathway plays a critical role in mediating this apoptotic cell death, leading to detrimental cardiac dysfunction. Significantly, our research expands the understanding of the complex mechanisms of PON-induced cardiotoxicity. We also provide strong evidence that BMP-7 can effectively counteract PON’s adverse effects by inhibiting pathways associated with apoptotic cell death. Notably, BMP-7 has been shown to reduce cardiac hypertrophy and fibrosis, while also restoring heart function. These findings lay a robust foundation for BMP-7’s potential to inhibit PON’s cardiotoxic side effects. While our study provides important preclinical insights, future work should focus on defining the long-term safety and optimal dosing of BMP-7 to advance its potential toward clinical translation. These could potentially benefit not only CML patients but also shed light on novel treatment strategies for different types of cardiotoxicities.

## Data Availability

Data is contained within the article.

## Supplementary Materials

The following supporting information can be downloaded at: https://www.mdpi.com/article/10.3390/ph18121776/s1, S1: Full Western blots.

## Abbreviations

The following abbreviations are used in this manuscript:

ABL	Abelson murine leukemia viral oncogene homolog
Akt	Protein kinase B
ANOVA	Analysis of variance
ApoE	Apolipoprotein E
AU	Arbitrary units
BAX	Bcl2-associated X-protein
Bcl2	B-cell lymphoma 2
BCR	Breakpoint cluster region
BMP-7	Bone morphogenetic protein 7
BSA	Bovine serum albumin
CML	Chronic myeloid leukemia
D19	Day 19
DAPI	4′,6-diamidino-2-phenylindole
EDV	End-diastolic volume
EF	Ejection fraction
ESV	End-systolic volume
FDA	Food and Drug Administration
FS	Fractional shortening
GAPDH	Glyceraldehyde-3-phosphate dehydrogenase
hiPSC-CMs	Human-induced pluripotent stem cell–derived cardiomyocytes
H&E	Hematoxylin and eosin
IACUC	Institutional Animal Care and Use Committee
IHC	Immunohistochemistry
IP	Intraperitoneal
IV	Intravenous
LV	Left ventricular
LVIDd	Left ventricular internal dimension-diastole
LVIDs	Left ventricular internal dimension-systole
MAPK	Mitogen-activated protein kinases
MOM	Mouse on mouse
NCI	National Cancer Institute
NFM	Non-fat milk
NGS	Normal goat serum
NIDDK	National Institutes of Diabetes and Digestive and Kidney Diseases
NIH	National Institutes of Health
pAkt	Phosphorylated Akt
PBS	Phosphate-buffered saline
PFA	Paraformaldehyde
PIP3	Phosphatidylinositol 3,4,5-trisphosphate
PON	Ponatinib
PTEN	Phosphatase and tensin homolog
PVDF	Polyvinylidene difluoride
RIPA	Radioimmunoprecipitation assay buffer
RT	Room temperature
Src-α-actin	Sarcomeric alpha-actin
SEM	Standard error of the mean
SMAD	Suppressor of mothers against decapentaplegic
tBID	BH3-interacting-domain death agonist
TBS-T	Tris-buffered saline, 0.1% Tween-20
TKIs	Tyrosine kinase inhibitors
TUNEL	Deoxynucleotidyl transferase dUTP nick end labeling
UCF	University of Central Florida
WB	Western blotting
