# Supplementary material for: BMP-7 Treatment Ameliorates PTEN-Akt Mediated Apoptosis and Adverse Cardiac Remodeling in Ponatinib-Induced Cardiotoxicity
Source: Pharmaceuticals (Basel). 2025 Nov 22;18(12):1776. doi: 10.3390/ph18121776 (PMC12736196; doi:10.3390/ph18121776)

# Figure 3 C Western Blot

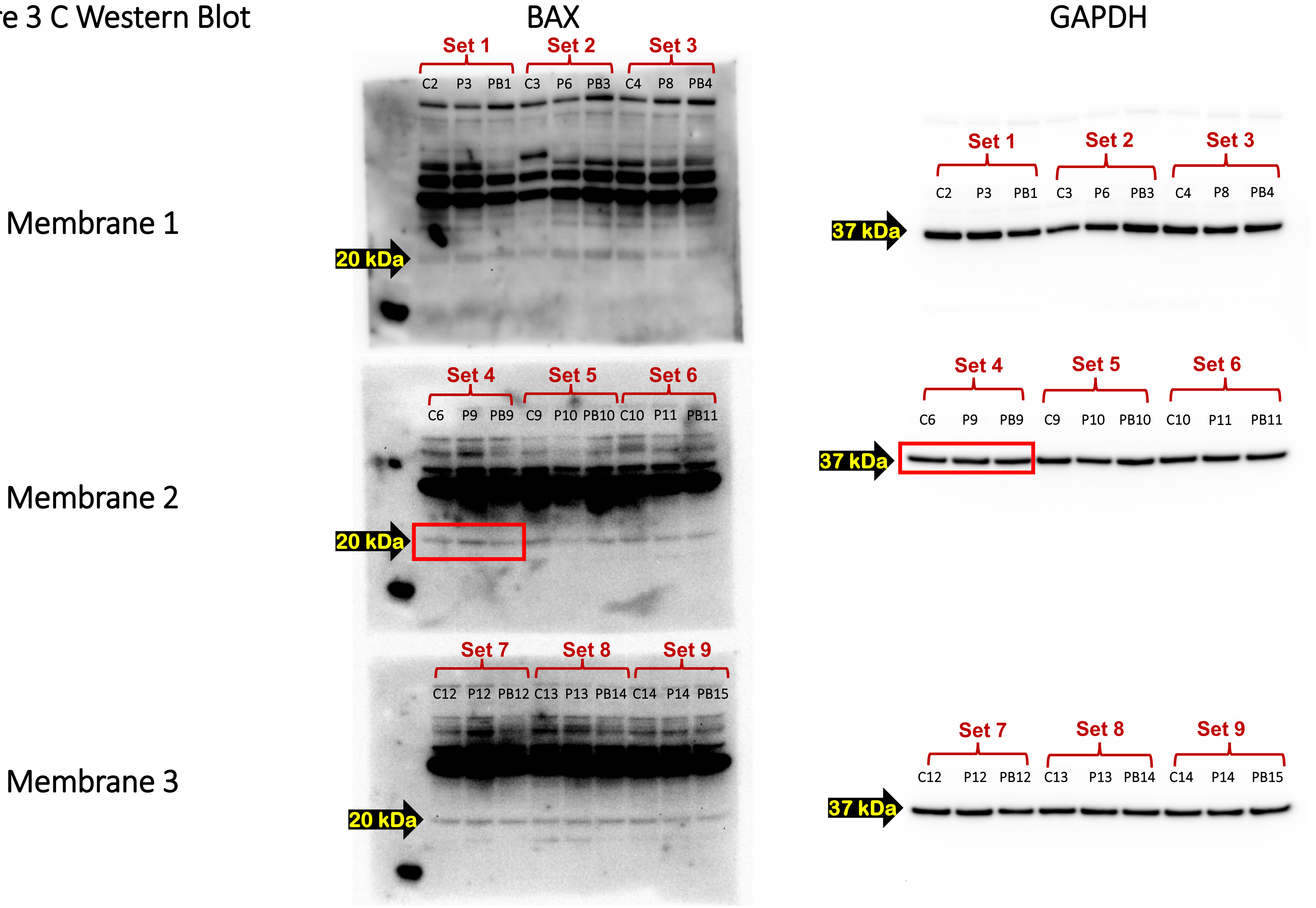

# Figure 4 C Western Blot

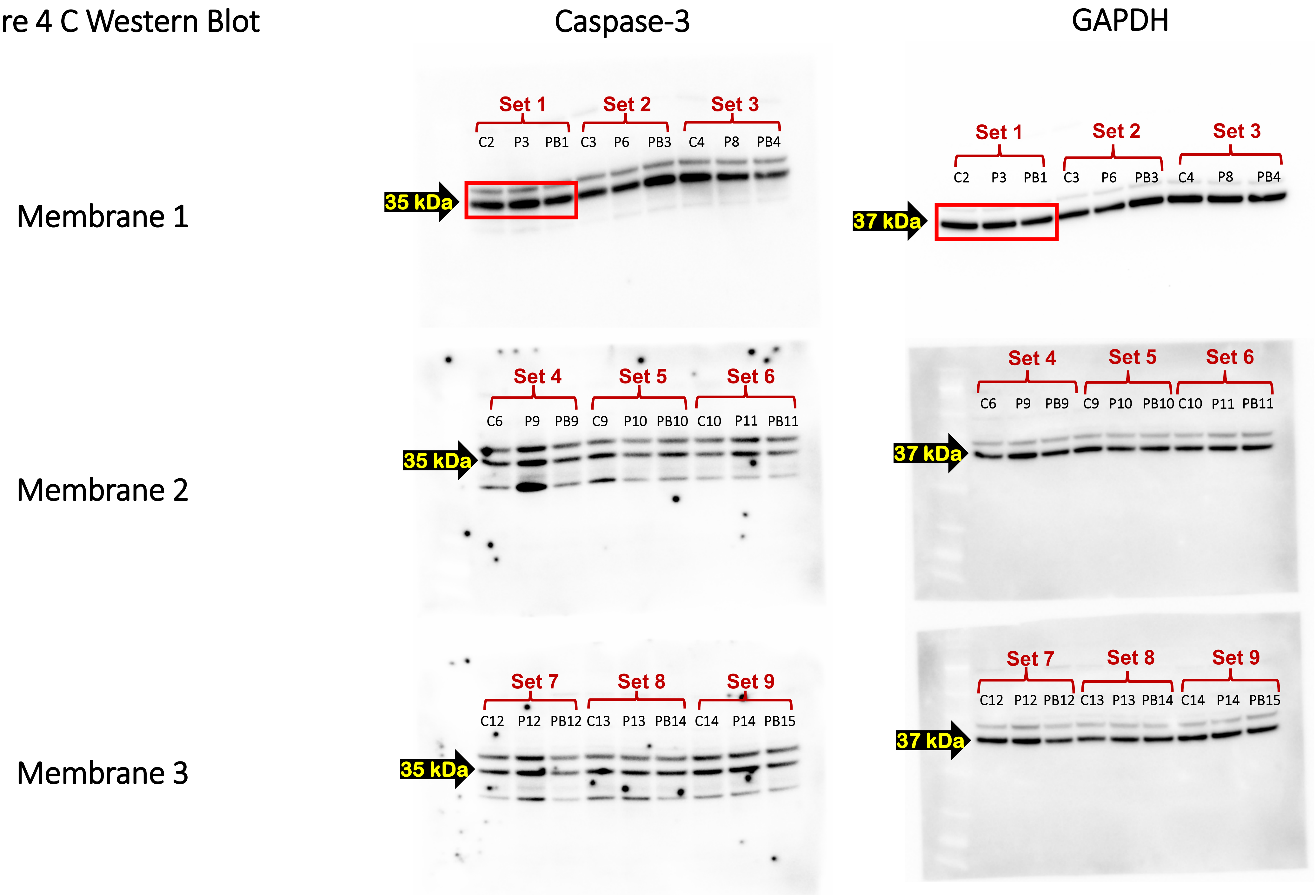

# Figure 5 C Western Blot

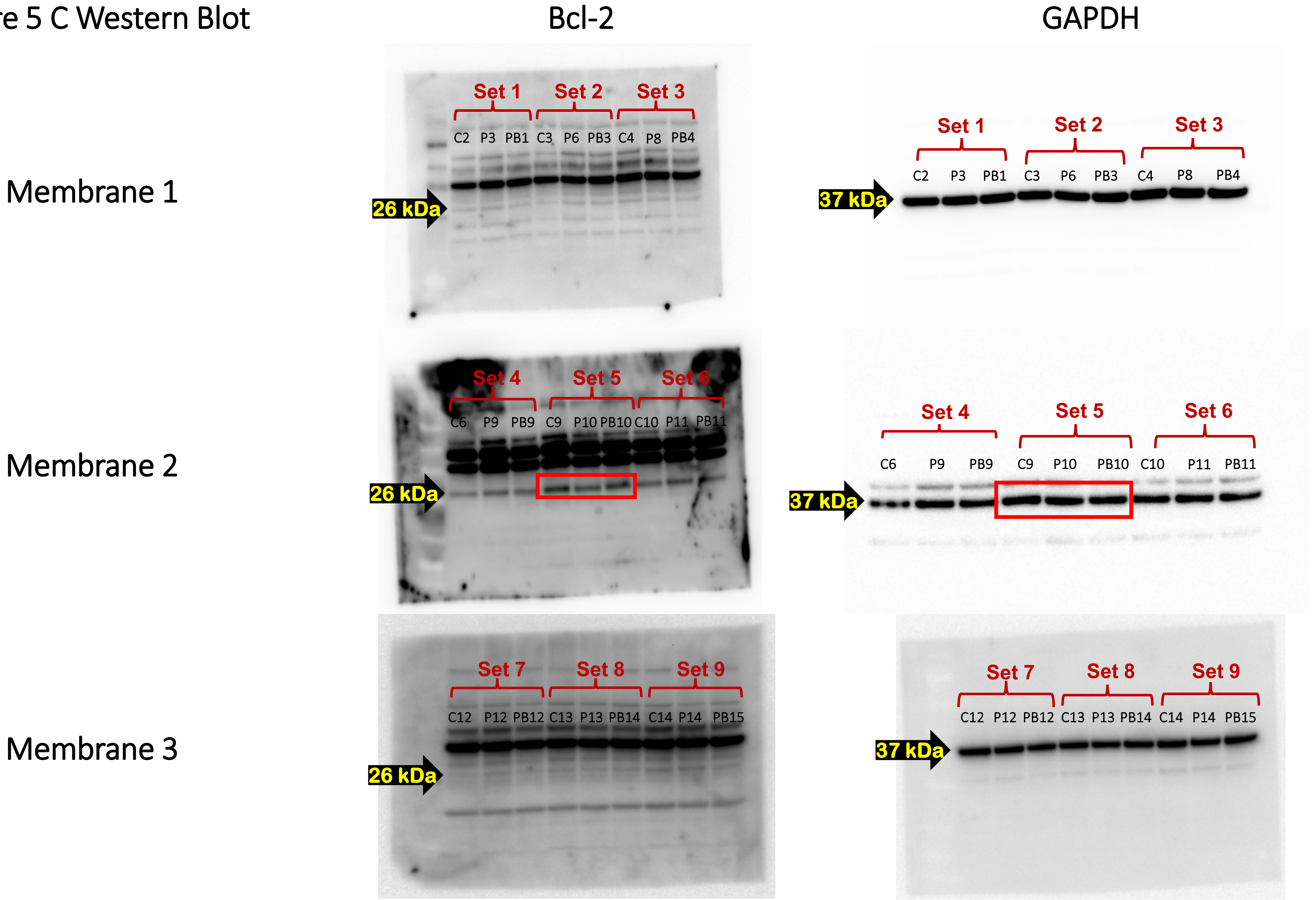

Figure 6 A Western Blot

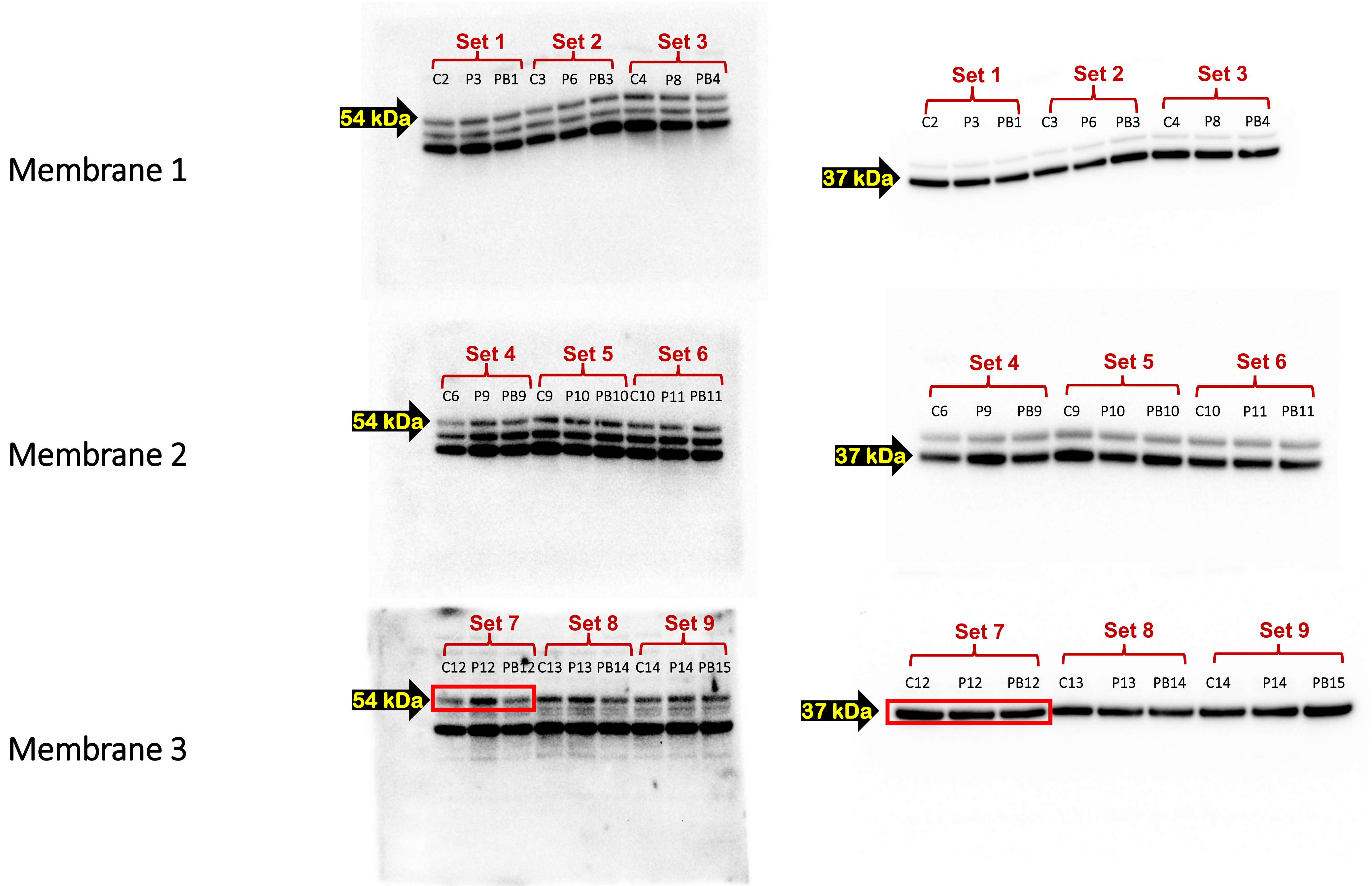

Figure 6 C Western Blot

pAKT GAPDH

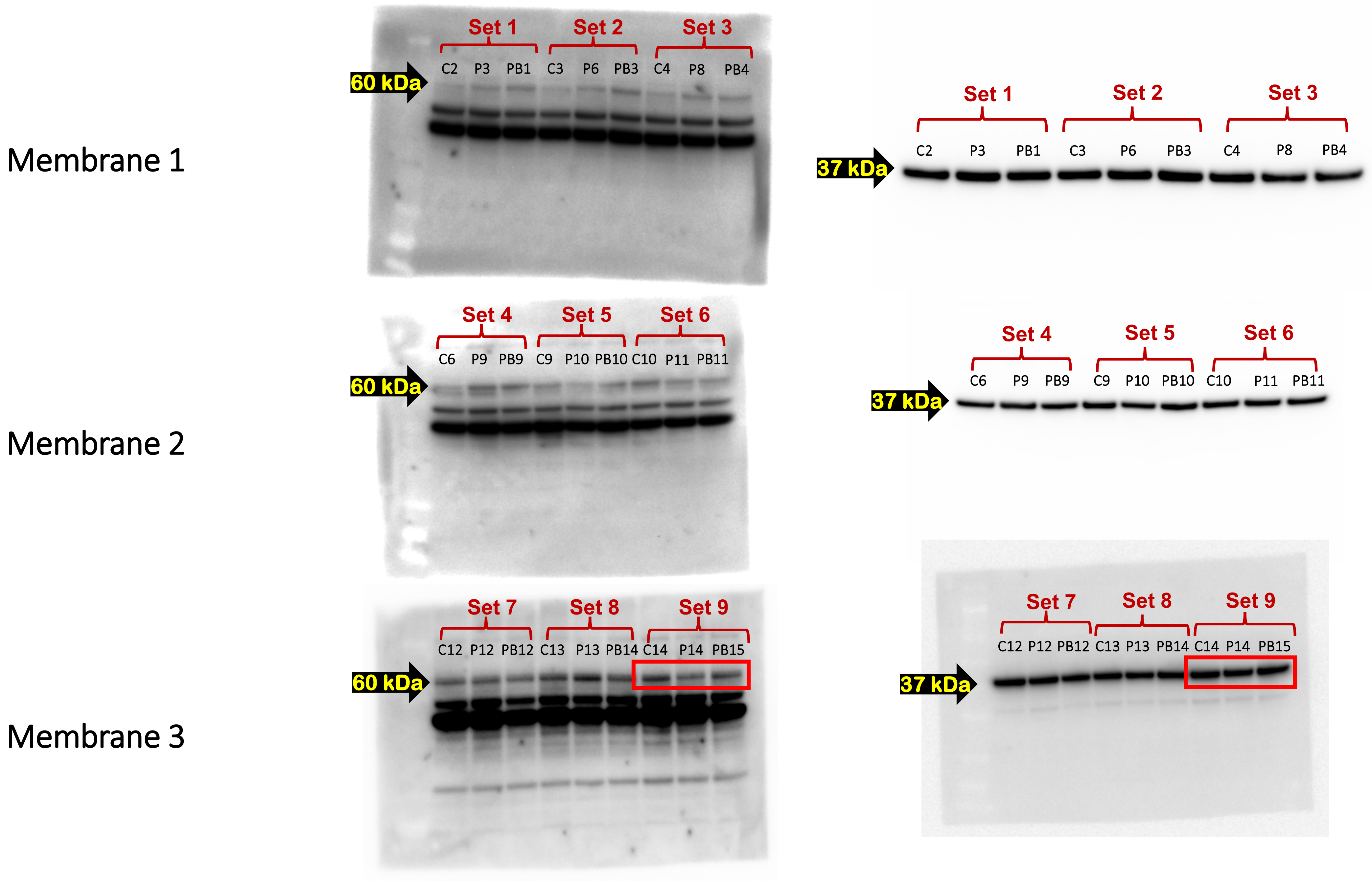

Supplement: Supplementary file 1 [file pharmaceuticals-18-01776-s001.zip › Supplementary Materials S1.pdf]
